# Immunotherapy in Renal Cell Carcinoma: The Future Is Now

**DOI:** 10.3390/ijms21072532

**Published:** 2020-04-05

**Authors:** Antoine Deleuze, Judikaël Saout, Frédéric Dugay, Benoit Peyronnet, Romain Mathieu, Gregory Verhoest, Karim Bensalah, Laurence Crouzet, Brigitte Laguerre, Marc-Antoine Belaud-Rotureau, Nathalie Rioux-Leclercq, Solène-Florence Kammerer-Jacquet

**Affiliations:** 1 Université Rennes, Inserm, EHESP (Ecole des Hautes Etudes en Santé Publique), IRSET (Institut de recherche en santé, environnement et travail), UMR 1085, 35000 Rennes, France; antoine.deleuze@chu-rennes.fr (A.D.); judikael.saout.1@univ-rennes1.fr (J.S.); frederic.dugay@chu-rennes.fr (F.D.); romain.mathieu@chu-rennes.fr (R.M.); marc-antoine.belaud-rotureau@chu-rennes.fr (M.-A.B.-R.); nathalie.rioux-leclercq@chu-rennes.fr (N.R.-L.); 2 Department of Medical Oncology, Centre Eugene Marquis, 35000 Rennes, France; l.crouzet@rennes.unicancer.fr (L.C.); b.laguerre@rennes.unicancer.fr (B.L.); 3 Department of Cytogenetics, University Hospital, 35000 Rennes, France; 4 Department of Urology, University Hospital, 35000 Rennes, France; benoit.peyronnet@chu-rennes.fr (B.P.); gregory.verhoest@chu-rennes.fr (G.V.); karim.bensalah@chu-rennes.fr (K.B.); 5 Department of Pathology, University Hospital, 35000 Rennes, France

**Keywords:** immunotherapy, immune checkpoint inhibitors, renal cell carcinoma, PD-1, PD-L1, ongoing trials, biomarkers, emerging drugs

## Abstract

Renal cell carcinoma is the third type of urologic cancer and has a poor prognosis with 30% of metastatic patients at diagnosis. The antiangiogenics and targeted immunotherapies led to treatment remodeling emphasizing the role of the tumour microenvironment. However, long-term responses are rare with a high rate of resistance. New strategies are emerging to improve the efficacy and the emerging drugs are under evaluation in ongoing trials. With the different treatment options, there is an urgent need to identify biomarkers in order to predict the efficacy of drugs and to better stratify patients. Owing to the limitations of programmed death-ligand 1 (PD-L1), the most studied immunohistochemistry biomarkers, and of the tumor mutational burden, the identification of more reliable markers is an unmet need. New technologies could help in this purpose.

## 1. Introduction

Renal cell carcinoma is the third urological cancer, representing 3% of all cancers in women and 5% in men with an incidence of around 400,000 cases worldwide [1]. The prognosis is poor: 30% of patients are metastatic at diagnosis and almost 30% of the remaining patients will develop metastases detected during the follow-up [2].

Clear cell renal cell carcinomas (ccRCC) represent the vast majority of RCC (around 75%). The other histologies mainly encompass papillary (20%) and chromophobe RCC (5%). The other entities are very rare including translocation-associated RCC, medullar RCC and collecting duct carcinoma. The histological entities based on distinct pathological features present different molecular alterations. Indeed, ccRCC are hallmarked by a frequent alteration of the *VHL* gene, a tumour suppressor gene, leading to angiogenesis through the transcription of genes regulated by *HIF* such as *VEGF* [3,4,5,6,7].

Non ccRCC (nccRCC) represent a heterogeneous group with papillary, chromophobe RCC and translocation RCC, the most frequent entities. Papillary RCC (pRCC) include tumours with indolent outcome (type 1) and more aggressive tumours (type 2) [8]. Type 1 and type 2 pRCC commonly harbor frequent *MET* alterations. However, alterations of *SETD2, CDKN2A, EGFR, NF2* and *TERT* have been described in type 2 and suggest the activation of MAP kinases pathway, cell cycle and deregulation of chromatin remodeling [9]. Chromophobe RCC (cRCC) are rarely metastatic and characterize mitochondrial alterations, frequently mutated *p53* and activation of mTOR pathway [9]. Translocation RCC (tRCC) harbor gene fusions involve *TFE3* and *TFEB*, members of the MiTF family [10]. These transcription factors have multiple partners, mainly involving messenger RNA splicing [11].

The other entities constitute less than 2% of renal tumours. The collecting duct carcinoma have been described as immunogenic tumours with high lymphocyte infiltration resultingfrom the upregulation of genes involved in T-cell activation and proliferation [12]. Renal medullary carcinoma present a frequent loss *INI1* (*SMARCB1*) implicated in the chromatin remodeling complex [13]. Among familial RCC syndromes, patients with hereditary leiomyomatosis and renal cell carcinoma (HLRCC) syndrome harbor *Fumarate Hydratase* (FH) germline mutation and develop clinical aggressive tumours [14]. The *FH* mutation by inactivating the enzyme alters the function of the Krebs cycle.

The sarcomatoid component can be found in all the histologic subtypes and demonstrates an increased tumour mutation burden (TMB) with high frequency of *p53*, *CDKN2A* and *NF2* mutations and also genes involved the chromatin remodeling such as *ARID1A* and *BAP1* [15].

The treatment and management of metastatic RCC have radically changed over the past 20 years [16]. Initially, first-generation immunotherapy with cytokines: interleukins or interferon represented standard approaches but with poor results [17,18]. The development of tyrosine kinase inhibitors, mainly vascular endothelial growth factor (VEGF) receptor inhibitors, largely improved the prognosis of both progression free survival (PFS) and overall survival (OS) [19].

The emergence of immune checkpoint inhibitors (ICI) alone or in combination (anti-cytotoxic T-lymphocyte antigen-4 (CTLA4) and anti-programmed death 1 (PD-1)) showed interesting results [20,21]. Targeted immunotherapy is an alternative to antiangiogenics because ccRCC is also considered an immunogenic tumour with high numbers of immune cells such as tumour-infiltrating lymphocytes (TIL) [22,23,24]. Recent trials proposed antiangiogenics in association with targeted immunotherapy to overcome resistance emphasizing the role of the tumour microenvironment (TME) and this strategy is currently an option in first line treatment [25,26].

Mechanisms of resistance with ICI can be primary or innate and secondary or acquired [27]. They encompass neo-antigen loss, defect of antigen presentation, alternative immune checkpoints and defective interferon signalling. Interferon-γ is a major mechanism of resistance by enhancing programmed death-ligand 1 PD-L1 expression and inducing the expression of immune inhibitory molecules [28]. Other immune checkpoints such as TIM-3, LAG-3 and TIGIT play a role in the resistance by inhibiting antitumour immune response [29]. Novel therapeutic approaches try to overcome these mechanisms of resistance and are under evaluation in ongoing trials.

Identifying biomarkers is the key to better select treatments, reduce costs and improve survival in patients with metastatic kidney cancer. However, the limitations of the most studied biomarkers: PD-L1 immunohistochemistry and TMB make necessary the identification of robust markers. New technologies could help in this purpose.

In this comprehensive review, we will discuss: 1. the specificities of the TME in RCC, 2. the treatment update with the results of recent trials, 3. the emerging drugs used in ongoing trials, 4. the predictive biomarkers and 5. the novel technologies.

## 2. Specificities of the Tumour Microenvironment in Renal Cell Carcinoma

### 2.1. Vascular Component

Angiogenesis has been described to play an important role in the progression of RCC and leads to the recruitment of endothelial cells. Recent data suggest that endothelial cells in TME differ from normal endothelial cells [30]. Akino et al. identified aneuploidy in one third of endothelial cells freshly dissociated from RCC [31]. This warrants further investigation as it could impact the response to antiangiogenic drugs. Moreover, Edeline et al. demonstrated two different angiogenic phenotype described as mature and immature. These two patterns could coexist within the same tumour demonstrating the heterogeneity of the vascular component [32]. Dufies et al. demonstrated in experimental tumours that sunitinib stimulated the development of lymphatic vessels. Indeed, these vessels are crucial for the recruitment of immune cells [33].

### 2.2. Immune Component

The expression of PD-L1 is widely represented in RCC suggesting the important role of PD-1/PD-L1 checkpoint with the aberrant expression of tumour cells. Indeed, PD-L1 expression was reported in 23% of ccRCC, 10% of pRCC, 5.6% of cRCC, 30% of t RCC and 20% in collecting duct carcinoma [34,35]. An amplification of 9p24.1, locus of PD-L1, was recently identify in RCC with sarcomatoid component leading to PD-L1 constitutive expression [36].

The immune compartment mainly include T cells, NK cells, B cells, macrophages and dendritic cells with complex interactions. Recently, Chevrier and colleagues used mass cytometry to compile an atlas of immune cells from 73 RCC identifying 22 T cell and 17 tumour-associated macrophage phenotypes with distinct immune composition between tumours [37]. Some macrophages such as M-11 or M-13 were associated with a worse prognosis and could constitute new targets. The phenotype of T cells (CD8+) regarding the expression of immune checkpoints (PD-1, LAG-3, Tim-3) identified the immune-regulated profile as more aggressive in a cohort of 40 RCC [38]. The role of B cells in the TME is unclear with both anti- and pro-tumoral effects. In ccRCC, the density of B cells identified by immunohistochemistry was associated with poor prognosis [39]. Moreover, the B cell signature was correlated with poor prognosis in The Cancer Genome Atlas (TCGA) ccRCC cohort. B cells could exert pro-tumoral functions through different mechanisms such as secretion of immune-regulatory cytokines that affect T cells and macrophages.

To understand the immunomodulation of nivolumab, Chouieri et al. explored the morphological and molecular changes of mRCC (*n* = 91) at screening and on treatment [40]. Immunohistochemical analysis revealed an increase in CD3+, CD8+ and CD4+ lymphocytes. No consistent change was observe in the expression of PD-L1 in tumour cells. Transcriptional analysis identified the up-regulation of genes stimulated by interferon γ with high levels of related chemokine in peripheral blood. A study case reported a histological complete response after nivolumab in metastatic ccRCC [41]. Only fibrotic changes and CD8+ lymphocytes were detected after treatment.

## 3. Treatment Update in Renal Cell Carcinoma

### 3.1. First-Line Treatment

**Figure 1:**
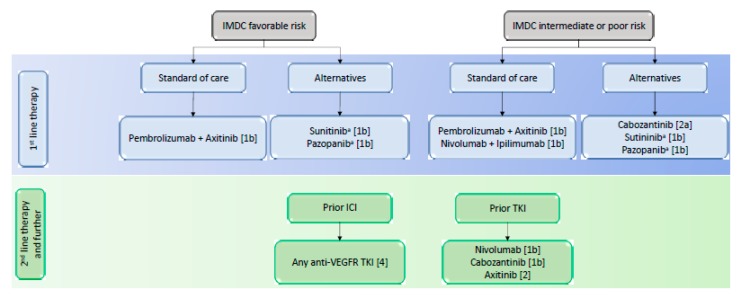
European Association of Urology Guidelines on Renal Cell Carcinoma. IMDC = International Metastatic Renal Cell CarcinomaDatabase Consortium; OS = overall survival; Oxford level of evidence: [1b] = based on one randomised controlled phase 3 trial; [2a] = based on one randomised controlled phase 2 trial; [2b] = subgroup analysis of a randomised controlled phase 3 trial; [4] = expert opinion. a = No OS benefit proven.

The ICI are the new backbone in the therapeutic landscape of renal cancer alongside tyrosine kinase inhibitors, Figure 1. Innovative combinations of ICI or ICI with TKI are now part of the treatment strategy and based on the results of recently published phase III trials in first line setting, Table 1 [20,25,26,42].

The Checkmate 214 trial confirmed the benefit of the nivolumab and ipilimumab association versus sunitinib in first line metastatic ccRCC among patients with intermediate or poor prognostic risk according to the International Metastatic Database Consortium (IMDC) risk model [20]. The objective response rate (ORR) was 42% versus 27%, and the complete response rate (CRR) was 11% versus 1% (*p* < 0.001). No differences were observed in terms of PFS. A recent update showed an interesting increase in OS after 30 months of follow-up in favor of nivolumab (anti-PD-1) plus ipilimumab (anti-CTLA4) combination (60% vs. 47%; HR: 0.66 ; CI 0.54 to 0.80 ; *p* < 0.0001) [43].

Recent updates also highlighted the benefit of ICI and TKI in combination. In Keynote 426, Pembrolizumab (anti-PD-1) plus axitinib showed a benefit in terms of OS at 12 month follow-up (90% vs. 78% ; HR: 0.53 ; CI: 0.38–0.74 ; *p* < 0.0001) leading to Food and Drug Administration (FDA) approval of the association in first line [26]. The Javelin 101 Renal comparing avelumab (anti-PD-L1) plus axitinib versus sunitinib in the PD-L1 positive population, defined as > 1% of positive immune cells staining within the tumour area, demonstrated a longer PFS, 13.8 months versus 8.4 months (HR = 0.69; 95% CI, 0.56 to 0.84; *p* < 0.001) and an improvement of the ORR (55.2% vs. 25%) [25]. After a 12 month follow-up, OS was not significantly different between the two arms. Similarly, the IMmotion 151 trial explored the atezolizumab (anti-PD-L1) and bevacizumab association regarding PD-L1 expression by immunohistochemistry (IHC) with a 1% cut-off on tumour-infiltrating immune cells [42]. The PFS was 11.2 months versus 8.4 months (HR = 0.74; 98.5% CI, 0.57 to 0.96; *p* = 0.02), but there was no statistical difference in OS at 24 months follow-up.

Among the recent phase III trials released, the distribution of patients in IMDC differed [20,25,26,42]. For instance, Checkmate 214 trial focused on intermediate- and poor-risk populations. The geography of recruitment may have an effect as the Keynote 426 trial had a large proportion of patients from outside the USA and Western Europe. Finally, RR and PFS are not strictly comparable. Moreover, follow-up remains short and it is difficult to formally conclude at this stage.

Considering the differences among trial populations and the lack of mature OS, the choice of first line therapy can be challenging. To date, guidelines for treatment decision rely on the IMDC risk model to stratify patients with untreated mRCC or previously treated with first-line targeted therapies [16,44]. However, the interest of this classification remains unclear with the advent of ICI combination therapies and patient characteristics stay major indicators in the absence of prospectively validated biomarkers. Indeed, a significant number of comorbidities could influence the treatment choice and should be confronted by the safety profile of ICI and TKI.

### 3.2. Second-Line Treatment

The choice of treatment in the second-line setting and beyond depends on the therapy previously received. In case of progression after antiangiogenics, the Checkmate 025 trial comparing nivolumab and everolimus showed an improvement of median OS in the nivolumab arm (25 vs. 19.6 months; HR = 0.73; 98.5% CI, 0.57 to 0.93; *p* = 0.0018) [45]. Notably, PD-L1 expression by IHC was associated with a poorer survival, but was not predictive of nivolumab efficacy. After first line ICI, any anti-angiogenic drug was recommended by the European Association of Urology with a low level of relevance [16]. However, due to the release of recent results, there is no prospectively validated data on the best therapeutic option after ICI and TKI combination in the front-line setting.

### 3.3. Adjuvant treatment

Finally, several ongoing phase III trials are studying the efficacy of ICI alone (atezolizumab, Pembrolizumab, nivolumab and tremelimumab) or in combination (nivolumab + ipilimumab) in the challenging peri-operative and adjuvant settings. Unsurprisingly, there is no combination of ICI plus TKI under evaluation as S-TRAC trial was the only one to show a benefit of adjuvant sunitinib in locally advanced high-risk ccRCC [46].

### 3.4. Non-Clear Cell Renal Cell Carcinoma

Unlike ccRCC, the management of nccRCC remains unclear and clinical trials are preferred [47]. Indeed, few prospective data for nccRCC treatment are available and trials have shown a lower efficacy of anti-angiogenic therapy compared to ccRCC with an impact on the prognosis of both PFS and OS [48]. In a recent retrospective trial including 41 patients, nivolumab seemed to demonstrate its efficacy with a favourable safety profile [49]. Indeed, the ORR was 20% and the median PFS was 3.5 months (95% CI; 1.9–5.0 months). Median OS was not reached, and the overall survival at the 10-month time point from the start of nivolumab treatment was 68%. Behind ccRCC, several trials evaluating ICI targeted drugs alone or in combination with TKI are currently recruiting in nccRCC without distinction of the entities, as shown in Table 2 and Table 3. Interestingly, the combination of durvalumab and savolitinib (MET TKI) is specifically evaluated in pRCC. Likewise, nivolumab plus axitinib is studied in tRCC. Of note, ongoing trials using emerging drugs or vaccinal strategies include de facto nccRCC under the non-restrictive RCC inclusion criteria, Table 4.

## 4. Emerging Drugs in Ongoing Trials Include Renal Cell Carcinoma

### 4.1. Inhibitory Immune Checkpoints

**Figure 2:**
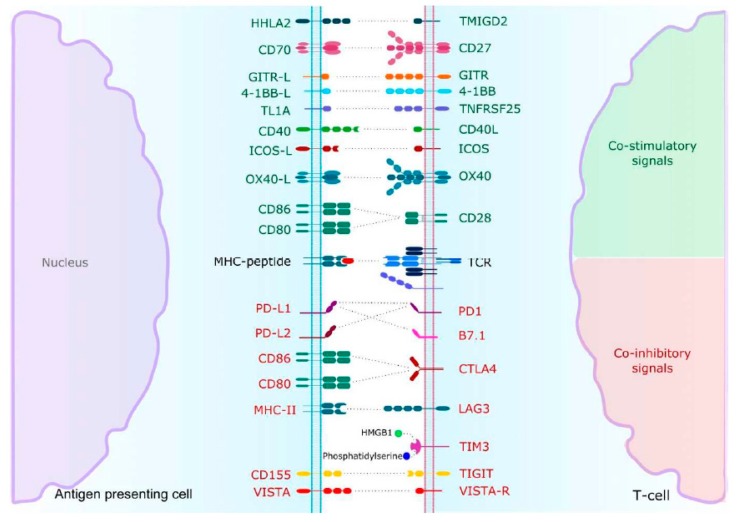
T-cell activation or inhibition is the result of an integrated and sequential intracellular signal cascade after MHC-peptide recognition by TCR. Abbreviations: GITR = Glucocorticoid-Induced TNF Receptor, ICOS = Inducible co-stimulator, OX40 = CD134, TCR = T-Cell Receptor, PD1 = Programmed Death 1, CTLA4 = Cytotoxic T-lymphocyte Antigen-4, LAG3 = Lymphocyte-associated gene 3, TIM3 = Tcell Immunoglobulin and Mucin domain-3, TIGIT = T-cell Immunoglobulin and ITIM domain, VISTA = Vdomain Immunoglobulin Suppressor of T-cell Activation.

The anergy of T cell is commonly understood as the result of a sequential transduction of signals implicating immune checkpoints in the immune synapse, Figure 2. If CTLA4 and PD-1 are the most common and well-studied, others are emerging and could be implied in resistance to traditional ICI and are evaluated in clinical trials including RCC, as shown in Table 2 and Table 3.

Lymphocyte-associated gene 3 (LAG3) is a transmembrane protein mainly expressed in activated T and natural killer (NK) cells, as shown in Figure 2 [29]. LAG3 is located on T cells. It shares a structural homology with CD4 and binds its ligand, the major histocompatibility complex class II (MHCII) with higher affinity. Moreover, the LAG3 blockade leads to an increased production of interferon gamma (INFγ), tumour necrosis factor alpha (TNFα) and pro-inflammatory interleukins [50].

Other ICI are emerging. Among them, T cell immunoglobulin and mucin domain3 (TIM3) expressed on a wide variety of immune cells. TIM3 contributes to immune tolerance by inhibiting T cells activation, mostly by upregulation of apoptosis [51]. Interestingly, there could be a synergistic effect with PD-1-PD-L1 blockade, reversing T cell exhaustion and improving anti-tumoral immune response [52].

T cell immunoglobulin and ITIM domain (TIGIT) is mainly found on TIL and is immunosuppressive through disrupting interleukins production and antigen-presenting cell (APC) maturation [53]. The two ligands Nectin-2 and CD155 are expressed in various cellular types from tumour to immune cells. Similar results in terms of T cells exhaustion were observed in the B7-H3 pathway [54].

The v-domain immunoglobulin suppressor of T cell activation (VISTA) is predominantly expressed on myeloid-derived suppressive cells (MDSC) and APC and down-regulates T cells activation [55]. Remarkably, VISTA blockade seemed to inhibit regulatory T cell immunosuppressive functions [56].

### 4.2. Co-Activating Immune Checkpoints

Tumour-specific T cell-mediated immune response is balanced by both inhibitory and co-stimulatory signals. If ICI are mainly used to restore immune response, agonist drugs are developed to increase co-stimulatory signals and stimulate immune response, as shown in Figure 2. Like ICI, several co-activating immune checkpoints are used in ongoing trials, Table 2 and Table 3.

On T cells, CD28 is known to deliver an activating signal when binding CD80/86 after TCR recognition of major histocompatibility complex (MHC). Inducible co-stimulator (ICOS) mainly located on CD4+ T cells belongs to the immunoglobulin family as well as CD28 and produces inflammatory cytokines. Its intracytoplasmic structure has a strong affinity for phosphoinositide 3-kinase (PI3K) favoring the proliferation signal in lymphocytes [57].

The tumour necrosis factor (TNF) receptor superfamily is represented by a group of both soluble and transmembrane receptors involved in inflammation processes and able to bind a variety of ligands such as TNFα, TNFβ and OX40 ligand [58]. When binding its ligand, OX40 promotes T cells proliferation and survival, particularly CD4+ and CD8+ T cells, by upregulating pro-inflammatory cytokines and anti-apoptotic molecules. Other member TNF receptors such as CD40, CD27 and 4-1BB contribute to increased cytotoxic T cells mediated response through apoptosis or memory-cell differentiation. The glucocorticoid-induced TNF receptor (GITR), located on CD4+ and CD8+ T cells and predominantly on FoxP3+ regulatory T cells, is known to enhance immunity to tumours through the attenuation of the effector activity of immunosuppressive regulatory T cells [59].

### 4.3. Metabolic Pathways

**Figure 3:**
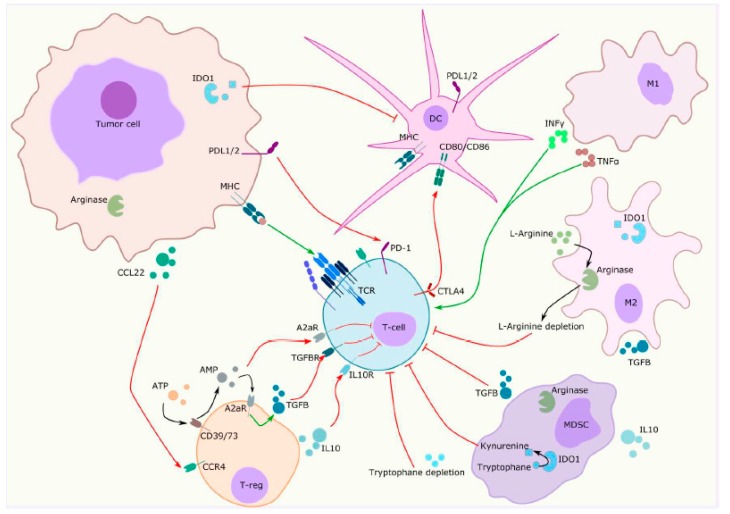
Tumor microenvironment is commonly defined as the co-existence of tumor cells interacting with resident and infiltrating host cells, secreted factors and extracellular matrix proteins. Among them, immunosuppressive cells are recruited in the tumor microenvironment by chemotaxis and are responsible for immunomodulatory cytokines production enhancement and decreased essential amino acids availability, resulting in favorable conditions for tumor growth. MDSC = Myeloïd-derived suppressive cells; T-cell = T lymphocyte; T-reg = Regulator lymphocyte; M2 = Type 2 macrophage; DC = dendritic cell; TGBß = tumor growth factor ß; A2aR = adenosine 2a receptor; ATP = adenosine triphosphate; AMP = adenosine monophosphate; IL10 = interleukin-10; MHC = major histocompatibility complex; PD1 = programmed death protein 1; PDL1 = programmed death ligand 1; IDO1 = indoleamine 2,3 dioxygenase 1; CTLA4 = cytotoxic T-lymphocyte antigen-4; CCL22 = C-C motif chemokine Ligand L22; CCR4 = C-C motif chemokine receptor 4; M1 = type 1 macrophage; INFγ = interferon γ; TNF α = tumor necrosis factor α.

Metabolic changes influence the TME by providing immunosuppressive metabolites and favoring tumour growth in hypoxic conditions, as shown in Figure 3 [60]. Multiple enzymes have been identified as key regulators of cytotoxic T cells immune response and are under evaluation in clinical trials, as shown in Table 2 and Table 3.

Among them, indoleamine 2,3 dioxygenase 1 (IDO1), an intracellular enzyme, catalyses the conversion of tryptophan into kynurenine [61]. Although physiologically expressed in stromal and dendritic cells, it is overexpressed in MDSC and tumour cells. The tryptophan depletion induced by IDO1 expression leads to T cell exhaustion and apoptosis. Moreover, high concentration of kynurenine promotes immune-tolerant dendritic cells and regulatory T cell proliferation [62].

Adenosine is a purine base known to bind G-protein coupled adenosine receptors, upregulated in activated immune cells [63]. Adenosine 2a receptor (A2aR) triggers upregulation of adenylate cyclase activity leading to increased cyclic adenosine monophosphate (cAMP) concentration. It has a profound immunomodulatory effect on immune cells by various mechanisms including ZAP70 inhibition in TCR signalling, IL2 down-regulation, FoxP3 expression enhancement on regulatory T cells and TGFß marked secretion [64].

As previously reported, TME acquires immunosuppressive features by reprogramming metabolic processes. Arginine is an essential amino acid in immune and tumour cells demonstrating a high level of arginase [65]. Arginase inhibitors are currently evaluated in combination with several ICI, Table 3.

### 4.4. Other Strategies

Various innovative strategies in immunotherapy are applied for RCC. The histone conformation impacts the transcription and is regulated through phosphorylation, sumoylation, ubiquitination, acetylation and deacetylation [66]. Histone deacetylase (HDAC) inhibitors modify the chromatin accessibility and play a crucial role in cycle arrest and apoptosis and could eventually enhance tumour antigens release and indirectly improve antigen presentation by APC and T cells priming [67]. In RCC, entinostat, panobinostat and chidamide are under evaluation in combination with ICI or TKI, as shown in Table 2 and Table 3.

Several vaccinal strategies are tested in cancers with the common objective to upregulate tumour neo-antigens exposure to immune system, particularly by T cells priming phase improvement [68]. At this time, three broad vaccine types are under investigation including DNA/RNA-based, peptide-based and cell-based vaccines with encouraging results in RCC. Oncolytic viruses are interesting alternatives, designed to infect tumour cells and hijack cellular machinery to induce transgene expression.

The chimeric Antigen Receptor (CAR) T cells are T cells genetically engineered to produce an artificial T cell receptor that combines both antigen-binding and T cell activating functions. The use of CART cells is hampered in RCC by the tumour heterogeneity. CAR T cells are currently tested in renal cell carcinoma, targeting various antigens such as ROR2, AXL, CD70, VEGFR2, MET or CAIX, as seen in Table 3. Moreover, pre-clinical data suggest the rationale of combining CAR T cells with TKI or radiotherapy [69].

## 5. Predictive Biomarkers in RCC

### 5.1. Clinico-Biological Biomarkers

The IMDC risk model includes six clinical and biological variables (poor Karnofsky performance status, less than 1 year between the diagnosis and the treatment, low hemoglobin concentration, high platelet count, high neutrophil count and high serum calcium) and has been validated in ccRCC and nccRCC [70,71]. However, the use of this classification is limited in nccRCC because the group is heterogeneous and samples in each group are small. The guidelines are dichotomized between favorable- and intermediate- or poor-risk disease, as shown in Figure 1 [16]. For instance, the clinical outcome was better in patients with intermediate- or poor-risk disease under nivolumab plus ipilimumab [20]. On the contrary, patients with favorable-risk disease better answered to sunitinib. These results suggest a distinct underlying biology.

### 5.2. Immunohistochemical Biomarkers

The most studied immunohistochemical biomarker PD-L1 failed to demonstrate a predictive capability in metastatic RCC [20]. If it was demonstrated as a poor prognostic factor in both ccRCC and nccRCC; indeed its ability to discriminate between patients the good responders is questionable [35]. Limitations to the use of PD-L1 have been well described and encompass intra-tumoral heterogeneity, variability of cut-offs and heterogeneous expression between primary and metastatic sites among others [72].

The contradictory and unresolved issue about PD-L1 as a biomarker also reveals the complicated interactions between the tumour and immune response, as shown in Figure 3. A comprehensive immune phenotype including other immunosuppressive factors such as TGFβ or IDO-1 and a better characterization of immune cells could be another step forward in the search of predictive biomarkers. Interestingly, IDO-1 expression was higher in endothelial cells of responders to nivolumab in a cohort of 15 patients with metastatic RCC [73]. This results in a reduced influx of tryptophan in the surrounding tumour tissue leading to a decrease in tumour proliferation.

### 5.3. Transcriptomic Analysis

Beuselink and colleagues first performed clustering transcriptomic analysis in patients with metastatic ccRCC (*n* = 53) receiving first-line sunitinib [74,75]. Hakimi et al. performed the same analysis in ccRCC of patients included in COMPARZ trial receiving pazopanib or sunitinib (*n* = 453) and identified four similar clusters [75]. Cluster 3 had the best prognosis with high angiogenic gene expression being associated with a better outcome under antiangiogenic therapy and similar to ccrcc2 cluster reported by Beuselinck et al. [76]. Of note, PBRM1 mutation was frequently associated with angiogenic gene expression. Cluster 4 had a worse prognosis and was similar to ccrcc4 with upregulation of immune pathways. Interestingly, ccrcc4 was enriched in tumours with sarcomatoid differentiation with a frequent expression of PD-L1 [77]. Clusters 1 and 2 were intermediate clusters with a lower expression of angiogenic and immune genes. A consensus classification could emerge from these different studies as proposed in the bladder cancer [78].

McDermott and colleagues explored three gene expression signatures: angiogenesis, T-effector/IFN-γ response, and myeloid inflammatory genes in ccRCC (*n* = 263) from IMmotion 150 trial: a phase 2 trial before IMmotion 151 of atezolizumab (anti-PD-L1) alone or combined with bevacizumab (anti-VEGF) versus sunitinib [76]. Interestingly, high T effector/IFNγ signature was associated with a better outcome to atezolizumab plus bevacizumab and a high myeloid inflammation signature was associated with reduced survival in the atezolizumab alone arm.

### 5.4. Tumour Mutational Burden and Mismatch Repair Deficiency

The TMB is based on the total number of mutations per coding area of the tumour genome. High mutation burden favors the formation of neo-antigens that enhance the tumour immune response [79]. This biomarker initially failed to identify responders to immunotherapy in ccRCC but is still under evaluation [80,81]. However, Limitations of this marker may be due to technical requirements such as coverage, DNA amount and analysis time and lack of standardization.

Even if RCC are not considered to be in the spectrum of hereditary non-polyposis colon cancer (HNPCC) or Lynch syndrome, loss of mismatch repair (MMR) proteins leading to microsatellite instability is frequently observed [82]. MMR deficient tumours demonstrate a higher rate of mutations but on specific genes. In RCC, data on the response to immunotherapy according to MMR status are also still ongoing.

### 5.5. Gut Microbiome

The gut microbiome, defined by the environmental conditions and the collection of microorganism and host genomes in an ecosystem, seems to influence the response to ICI. Indeed, the use of antibiotics could unfavorably impact the response. Indeed, Derosa et al. demonstrated a shorter survival and higher rates of progressive disease in patients with mRCC who received antibiotics within the month following the beginning of the treatment [83]. Indeed, antibiotics-related dysbiosis impacted the mirobiome and could decrease the activity of ICI. Conversely, they identified that some bacteria such as *B. salyersiae* or *A. muciniphila* could restore the efficacy of ICI.

## 6. Future Directions

### 6.1. Microenvironment Cell Population Counter

The microenvironment cell population counter (MCP counter) aims to assess the proportion of immune and stromal cells in the TME from transcriptomic data [84]. They classified tumours into four molecular TME subgroups: immune infiltration, T and NK lymphocytes, MCH1 expression and fibroblastic infiltration. This classification could be judiciously applied to previous transcriptomic data.

### 6.2. Single-Cell Technologies

The development and proper use of immunotherapies rely on the detailed understanding of tumour composition. However, this purpose is hampered by intratumour heterogeneity, which was demonstrated to be extensive in ccRCC [85]. With recent developments in single-cell technologies though, we can now circumvent and integrate intratumour heterogeneity into analyses in oncology by characterizing each individual cells within tumours [86]. In RCC, Kim and collaborators performed single-cell RNA sequencing to study the intratumour heterogeneity of paired primary RCC and lung metastasis [87]. A considerable variability between the primary and metastatic sites and among tumour cells was demonstrated by the activation of drug target pathways. This study encourages the use of single-cell RNA sequencing to better characterize cell populations and to favor the discovery of new biomarkers.

### 6.3. 3D Culture Models

The 3D culture models of ccRCC could represent good pre-clinical models based on tissue slices preserving the stromal components of the TME [88]. Already used for TKI targeted therapies, these models are increasingly being considered as potential platforms for monitoring immunotherapy responses [89]. Recent studies indeed reported an active immune TME after applying immunomodulatory molecules on tumour slice organotypic cultures, opening the way for their study on 3D ex vivo tumour models [90]. Interestingly, co-cultures of tumour-derived organoids were enriched with lymphocytes, leading Dijkstra and collaborators to recently demonstrate the generation of tumour-reactive T cells by co-culture of peripheral blood lymphocytes and colorectal cancer-derived organoids [91]. These precursor approaches will allow to learn more about immune escape mechanisms and determine associated predictive biomarkers.

### 6.4. Imaging

Novel methods of imaging are non-invasive and assess RCC at different time points. Bensch et al. demonstrated that positron emission tomography (PET) imaging was able to localize ^89^Zr-labeled atezolizumab to tumours expressing PD-L1 between primary and metastatic sites [92]. The uptake in tumours was heterogeneous and the clinical response better correlated with the pretreatment PET signal than with PD-L1 immunohistochemistry or T-effector gene expression signature. Moreover, CD8+ T cell infiltration was inferred from computed tomography [93].

### 6.5. Circulating Tumour Cells

The circulating tumour cells (CTC) is a non-invasive method isolating CTC from blood circulation [94]. The challenge is to detect them because of the rare expression of the usual marker epithelial cell adhesion molecule (EpCAM). This is due to the transdifferentiation of tumour cells through the epithelial mesenchymal transition [95]. The antibodies directed against membrane carbonic anhydrase 9 (CA9) and CD147 largely improved the detection of CTC from 17% with EpcAM to 97% of samples with CA9 and CD147 markers [96]. Other methods are based on RT-PCR targeting VHL gene alteration and size-based blood filtration associated with genetic and morphological analyses [97]. The CTC analyses could be used to select patients for clinical trials guided by biomarkers. The phenotype could evolve under treatment and be timely investigated to adapt the therapeutic strategy.

## 7. Conclusions

Renal cell carcinoma include distinct entities with specific molecular alterations. Among them, ccRCC, the most frequent, is particularly characterized by its angiogenic and immunogenic TME with complex interactions between stromal and immune cells. Like tumour cells, intratumour heterogeneity is present in the TME with a variable distribution and phenotype and consequently favours resistance to treatment.

Therapeutic options for patients with mRCC have expanded rapidly over the past decade with targeted immunotherapy being the new corner stone. Several emerging drugs are designed to enhance the antitumour immune response and are tested in ongoing trials.

With an increasing number of treatment options available, improved biomarkers are needed to better stratify patients and define the optimal selection of patients and the sequence of treatment to overcome resistance. Promising biomarkers such as gene expression signatures or gut microbiome are under evaluation. It is likely that the future of predictive biomarkers relies on the combination of different approaches reflecting the complexity of the TME.

We hope that, in the future, new technologies such as single-cell technologies contribute to unravel the intratumour heterogeneity, identify predictive biomarkers and discover new treatment targets.

## Figures and Tables

**Table 1:** Comparison of pivotal phase III clinical trials with available results evaluating immune checkpoints inhibitors.

Study Name	Tested Drugs	Comparison	Phase	Histology	Therapy Setting	OS (HR, 95% CI, *p*)	Median PFS (HR, 95% CI)	ORR (%)	CR (%)	Grade 3 (%)
Javelin Renal 101	avelumab + axitinib	sunitinib	III	ccRCC	First line	12-mo: 86% vs. 83% (0.78; 0.55–1.08; *p* = 0.14)	13.8 vs. 7.2 mo (0.61)	55.2 vs. 25,5	3.4 vs. 1.8	71.2 vs. 71.5
Keynote 426	pembrolizumab + axitinib	sunitinib	III	ccRCC	First line	12-mo: 90% vs. 78% (0.53; 0.38–0.74; *p* < 0.0001)	15.1 vs. 11.1 mo (0.69; 0.57–0.84)	59.3 vs. 35.7	5.8 vs. 1.9	75.8 vs. 70.6
CheckMate 214	nivolumab + ipilimumab	sunitinib	III	ccRCC	First line	30-mo : 60% vs, 47% (0.66; 0.54–0.80; *p* < 0.0001)	11.6 vs. 8.4 mo (0.82; 0.64–1,05)	42 vs. 29	9 vs. 1	47 vs. 64
Immotion 151	atezolizumab + bevacizumab	sunitinib	III	ccRCC	First line	24 mo : 63% vs. 60% (0.93; 0.76–1.14; *p* = 0·4751)	11.2 vs. 7.7 mo (0.74; 0.57–0.96)	43 vs. 25	9 vs. 4	40 vs. 54

Abbreviations: OS = overall survival; HR = Hazard Ratio; PFS = progression-free survival; ORR = objective response rate; CR = complete response; ccRCC: clear cell renal cell carcinoma, mo = months.

**Table 2:** Clinical trials evaluating immune checkpoint inhibitors in association with tyrosine kinase inhibitors.

NCT number	Targeting Agents	Comparison	Phase	Histology	Primary Endpoint	Therapy Setting	Status
NCT03260894	pembrolizumab + epacadostat	sunitinib or pazopanib	III	ccRCC	ORR	First line	Active not recruiting
NCT02811861	lenvatinib + everolimus or pembrolizumab	sunitinib	III	ccRCC	PFS	First line	Active not recruiting
NCT03141177	nivolumab + cabozantinib	sunitinib	III	ccRCC	PFS	First line	Active not recruiting
NCT03793166	nivolumab, ipilimumab, cabozantinib	nivolumab or nivolumab + cabozantinib	III	ccRCC	OS	First line	Recruiting
NCT03937219	nivolumab + ipilimumab + cabozantinib	nivolumab + ipilimumab + placebo	III	ccRCC	DFS	First line	Recruiting
NCT03680521	sitravatinib + nivolumab	_	II	ccRCC	ORR	First line	Recruiting
NCT02960906	nivolumab, ipilimumab, VEGFR-TKI	_	II	ccRCC	ORR	First line	Recruiting
NCT03736330	axitinib + pembrolizumab + D-CIK	_	II	ccRCC	ORR	At least second line	Recruiting
NCT02819596	savolitinib, durvalumab, tremelimumab	_	II	ccRCC, pRCC	DLT, ORR	At least second line	Unknown
NCT02964078	pembrolizumab + interleukin-2	_	II	ccRCC	ORR	Any	Active not recruiting
NCT03092856	anti-OX40 agonist antibody + axitinib	_	II	ccRCC	PFS	No standard anymore available	Recruiting
NCT02724878	atezolizumab + bevacizumab	_	II	nccRCC	ORR	Any	Active not recruiting
NCT03635892	nivolumab + cabozantinib	_	II	nccRCC	ORR	Any	Recruiting
NCT03595124	nivolumab + axitinib	_	II	tRCC	PFS	Any	Recruiting
NCT02493751	avelumab + axitinib	_	I/II	ccRCC	DLT	First line	Published
NCT02899078	ibrutinib + nivolumab	_	I/II	ccRCC, nccRCC	PFS	At least second line	Recruiting
NCT02348008	pembrolizumab + bevacizumab	_	I/II	ccRCC	Safety, efficacy	At least second line	Active not recruiting
NCT03172754	nivolumab + axitinib	_	I/II	ccRCC	Safety	At least second line	Recruiting
NCT03024437	atezolizumab + entinostat + bevacizumab	atezolizumab + entinostat	I/II	ccRCC	Safety, ORR	At least second line	Recruiting
NCT02501096	pembrolizumab + lenvatinib	_	I/II	ccRCC	DLT, ORR	No standard anymore available	Active not recruiting
NCT01472081	nivolumab + sunitinib or pazopanib	nivolumab	I	ccRCC	Safety	At least second line	Published
NCT03307785	TSR-022 (anti-TIM3)/niraparib/TSR-042 (anti-PD1)/chemotherapy/bevacizumab	_	I	RCC and others	Safety	At least second line	Active not recruiting
NCT03200587	avelumab + cabozantinib	_	Ib	ccRCC	Safety	Any	Recruiting

Abbreviations: OS = overall survival; HR = Hazard Ratio; PFS = progression-free survival; ORR = objective response rate; CR = complete response; ccRCC = clear cell renal cell carcinoma; nccRCC = non clear cell renal cell carcinoma; DLT = dose-limiting toxicity; DFS = disease-free survival; ICI = immune checkpoint inhibitor; TKI = tyrosine kinase inhibitor; VEGFR: vascular endothelial growth factor receptor; _ = na; SBRT = stereotactic body radiation.

**Table 3:** Clinical trials evaluating targeted immunotherapies alone or in combination.

NCT Number	Targeting Agents	Comparison	Phase	Histology	Primary Endpoint	Therapy Setting	Status
NCT03729245	NKTR-214 (IL2R agonist) + nivolumab	sunitinib or cabozantinib	III	ccRCC	ORR, OS	First line	Recruiting
NCT03873402	nivolumab + ipilimumab	nivolumab	III	ccRCC	PFS, ORR	First line	Recruiting
NCT01668784	nivolumab	everolimus	III	ccRCC	OS	At least second line	Published
NCT03055013	nivolumab	observation	III	ccRCC	DFS	Peri-operative	Recruiting
NCT03024996	atezolizumab	placebo	III	ccRCC	DFS	Adjuvant	Active not recruiting
NCT03138512	nivolumab + ipilimumab	placebo	III	ccRCC	DFS	Adjuvant	Recruiting
NCT03142334	pembrolizumab	placebo	III	ccRCC	DFS	Adjuvant	Active not recruiting
NCT03288532	nivolumab, tremelimumab	_	III	ccRCC	DFS	Adjuvant	Recruiting
NCT02996110	BMS-986205 (IDO1 oral inhibitor) +/− nivolumab +/− ipilimumab	nivolumab +/− ipilimumab	II	ccRCC, nccRCC	ORR, PFS	First line	Recruiting
NCT03552380	nivolumab + ipilimumab + entinostat	_	II	ccRCC	Safety	At least second line	Recruiting
NCT03501381	entinostat + IL2	IL2	II	ccRCC	PFS	At least second line	Recruiting
NCT03469713	nivolumab + SBRT	_	II	ccRCC	ORR	At least second line	Recruiting
NCT03177239	nivolumab + ipilimumab	_	II	nccRCC	ORR	Any	Active, not recruiting
NCT04262375	oleclumab (anti-CD73 antagonist mAb) + durvalumab	_	II	RCC and others	ORR, PFS	Any	Not yet recruiting
NCT03207867	NIR178 (A2aR antagonist) + spartalizumab (anti-PD1)	_	II	RCC and others	ORR	At least second line	Recruiting
NCT03693612	GSK3359609 (anti-ICOS) + tremelimumab	chemotherapies	II	RCC and others	DLT	No standard anymore available	Recruiting
NCT03693612	anti-ICOS + tremelimumab	_	II	RCC and others	Safety, DLT	No standard anymore available	Recruiting
NCT01038778	entinostat + aldesleukin	_	I/II	ccRCC	Dose, ORR	At least second line	Active, not recruiting
NCT03308396	durvalumab + guadecitabine	_	I/II	ccRCC	Safe dose/ORR	At least second line	Recruiting
NCT02989714	nivolumab + interleukin-2	-	I/II	ccRCC	Safety	Third line	Active not recruiting
NCT02460224	LAG525 (anti-LAG3) + spartalizumab (anti-PD1)	_	I/II	RCC and others	DLT, ORR	At least second line	Active, not recruiting
NCT03652077	anti-TIM3	_	I/II	RCC and others	Safety	No standard anymore available	Recruiting
NCT02608268	MBG453 (anti-TIM3) + spartalizumab (anti-PD1)	spartalizumab anti-PD1	I/II	RCC and others	Safety, ORR, DLT	No standard anymore available	Recruiting
NCT01968109	relatlimab (anti-LAG3) + nivolumab	relatlimab	I/II	RCC and others	Safety, ORR, DLT	No standard anymore available	Recruiting
NCT03126110	INCAGN01876 (anti-GITR) + nivolumab + ipilimumab	_	I/II	RCC and others	Safety, tolerability	No standard anymore available	Recruiting
NCT02335918	varlilumab (anti-CD27) + nivolumab	_	I/II	RCC and others	DLT, ORR	At least second line	Completed
NCT02718066	HBI-8000 (HDACi) + nivolumab	_	I/II	RCC and others	RP2D	At least second line	Recruiting
NCT02890069	spartalizumab + LCL16 (IAP inhibitor) + everolimus + panobinostat	_	I/II	RCC and others	DLT	At least second line	Recruiting
NCT02771626	CB-839 (glutaminase inhibitor) + nivolumab	_	I/II	RCC and others	Safety, efficacy	At least second line	Recruiting
NCT02817633	TSR-022 (anti-TIM3)/TSR-042 (anti-PD1)/TSR-033 (anti-LAG3)	_	I	RCC and others	Safety, tolerability	No standard anymore available	Recruiting
NCT03119428	OMP-31M32 (anti-TIGIT) + nivolumab	_	I	RCC and others	DLT	No standard anymore available	Terminated
NCT00351949	IMP321 (anti-LAG3)	_	I	RCC and others	Safety, tolerability	No standard anymore available	Completed
NCT02386111	varlilumab (anti-CD27)	_	I	RCC and others	Safety, tolerability	At least second line	Terminated
NCT03343613	LY3300054 (IDO1 inhibitor) + PD-L1 inhibitor	_	I	RCC and others	DLT	No standard anymore available	Recruiting
NCT02812875	CA-170 (VISTA antagonist)	_	I	RCC and others	DLT	No standard anymore available	Active not recruiting
NCT02655822	ciforadenant (A2aR antagonist) +/- atezolizumab	_	I	RCC and others	DLT	At least second line	Recruiting
NCT04198766	anti-OX40 + pembrolizumab	_	I	RCC and others	Safety	No standard anymore available	Recruiting

Abbreviations: OS = overall survival; HR = Hazard Ratio; PFS = progression-free survival; ORR = objective response rate; CR = complete response; ccRCC = clear cell renal cell carcinoma; nccRCC = non clear cell renal cell carcinoma; DLT = dose-limiting toxicity; DFS = disease-free survival.

**Table 4:** Vaccinal strategies and CAR T-cells trials.

NCT Number	Targeting Agents	Comparison	Phase	Histology	Primary Endpoint	Therapy Setting	Status
NCT00458536	dendritic cell tumor fusion vaccine + GM-CSF	_	I/II	ccRCC, nccRCC	Safety	Any	Active not recruiting
NCT03633110	GEN-009 Adjuvanted Vaccine + nivolumab + pembrolizumab	_	I/II	RCC and others	Safety	At least second line	Recruiting
NCT00722228	Autologous or Allogeneic tumor cells	_	I/II	RCC and others	Safety, efficacy	At least second line	Recruiting
NCT03393936	anti-ROR2 CAR-T or anti AXL CART-T	_	I/II	ccRCC, nccRCC	Safety	No standard available	Recruiting
NCT02830724	anti-CD70 CAR-T	_	I/II	RCC and others	Safety	At least second line	Recruiting
NCT01218867	anti-VEGFR2 CAR-T	_	I/II	RCC and others	ORR	At least second line	Terminated
NCT03638206	anti-C-MET CAR-T	_	I/II	RCC and others	Safety	At least second line	Recruiting
NCT02950766	neovax vaccine + ipilimumab	_	I	ccRCC	DLT	At least second line	Recruiting
NCT00096629	PSMA DNA vaccine	_	I	ccRCC, nccRCC	Safety	Adjuvant	Completed
NCT03548467	VB10.NEO vaccine +/- bempegaldesleukin (NKTR-214)	_	I	RCC and others	Safety	At least second line	Recruiting
NCT03294083	pexastimogene devacirepvec (Pexa-Vec)	_	I	ccRCC, nccRCC	Safety, efficacy	At least second line	Recruiting
NCT03715985	EVAX-01-CAF09b (peptide-based vaccine) +/- anti-PD1 or anti-PD-L1	_	I	RCC and others	Safety, efficacy	First line	Recruiting

Abbreviations: OS = overall survival; HR = Hazard Ratio; PFS = progression-free survival; ORR = objective response rate; CR = complete response; ccRCC = clear cell renal cell carcinoma; nccRCC = non clear cell renal cell carcinoma; DLT = dose-limiting toxicity; DFS = disease-free survival; ICI = immune checkpoint inhibitor; TKI = tyrosine kinase inhibitor; VEGFR: vascular endothelial growth factor receptor; - = na; SBRT = stereotactic body radiation.
